# A rare case report of tenosynovial chondromatosis of the semimembranosus-medial collateral ligament bursa

**DOI:** 10.1186/s12891-023-06337-6

**Published:** 2023-04-01

**Authors:** Cornelia Peterson, Minh Quan Le, Nathan D. McClain, Elena Ghotbi, Shadpour Demehri, John M. Gross, Mohammed Emam, John H. Wilckens

**Affiliations:** 1 Department of Molecular & Comparative Pathobiology, The Johns Hopkins University School of Medicine, Baltimore, MD USA; 2 Department of Comparative Pathobiology, Cummings School of Veterinary Medicine, Tufts University, North Grafton, MA USA; 3 Department of Physical Medicine & Rehabilitation, The Johns Hopkins Hospital, Baltimore, MD USA; 4 Department of Physical Therapy, The Johns Hopkins Hospital, Baltimore, MD USA; 5 Department of Radiology & Radiological Science, The Johns Hopkins Hospital, Baltimore, MD USA; 6 Department of Pathology, The Johns Hopkins University School of Medicine, Baltimore, MD USA; 7 Department of Orthopaedic Surgery, The Johns Hopkins Hospital University School of Medicine, 4924 Campbell Boulevard, White Marsh, Baltimore, MD USA

**Keywords:** Knee, Semimembranosus-medial collateral ligament bursa, Tenosynovial chondromatosis

## Abstract

**Background:**

Synovial chondromatosis is an uncommon metaplastic process of the synovial lining that results in the formation of cartilaginous nodules within joints or their associated bursae or tendon sheaths. Radiologic evidence of mineralized bodies within these structures is typically pathognomonic for this condition. Extraarticular chondromatosis is rarer than intraarticular chondromatosis, and the knee is affected less frequently than the smaller joints of the hands and feet. To our knowledge, no reports describing this condition in the semimembranosus-medial collateral ligament (SM-MCL) bursa have been published.

**Case presentation:**

We describe a case of tenosynovial chondromatosis in a 37-year-old woman. The case was atypical for both the location within the SM-MCL bursa and the paucity of radiodense or hypointense changes to support a clinical suspicion of chondroid metaplasia on radiographs and T2-weighted MRI, respectively. Recreational weightlifting and swimming by the patient were impaired by chronic pain, and restricted range of motion of the ipsilateral knee persisted despite extensive skilled physical therapy and injections of both corticosteroids and platelet-rich plasma. Thirteen months after a diagnostic and therapeutic knee arthroscopy, open surgical excision of the SM-MCL bursal body was performed, and knee pain and range of motion improved by the 6-week postoperative reevaluation. Pathologic evaluation of the excised tissue was consistent with tenosynovial chondromatosis.

**Conclusions:**

Synovial chondromatosis should be considered in the differential diagnosis for recalcitrant bursitis, even in the absence of classic imaging findings.

**Level of evidence:**

4.

## Introduction

The semimembranosus-medial collateral ligament (SM-MCL) bursa, also historically referred to as the semimembranosus-tibial collateral ligament bursa, is an extracapsular, U-shaped structure with a superficial portion wrapping around the distal aspect of the central semimembranosus tendon and a deep component located between the tendon and the underlying medial tibial condyle [1–3]. The SM-MCL is distinct from three other bursae of the medial knee, including the semimembranosus-gastrocnemius bursa, the pes anserine, and the medial collateral ligament bursa [4, 5]. The semimembranosus-gastrocnemius bursa is commonly referred to as a Baker’s cyst or popliteal cyst, when pathologically distended through an acquired intra-articular communication with the knee [6–8]. A distinct feature of the SM-MCL bursa is its non-communication with the joint. Onishi et al. [9] used ultrasound-guided latex injections to identify the SM-MCL bursa in cadaveric knees and further demonstrated upon gross dissection that injectate did not extend into the intra-articular space. Finally, the pes anserine, located interiorly and anteriorly to the SM-MCL, is another common source of medial knee pain [8].

Synovial chondromatosis is a benign, typically monoarticular, metaplastic condition that occurs uncommonly in intraarticular synovium and rarely in extraarticular tendon sheaths and bursae [10–14]. The pathophysiological characteristics of primary synovial chondromatosis are poorly elucidated; however, the presence of ectopic intra- or periarticular cartilaginous nodules in secondary synovial chondromatosis results from the pathologic mechanical sequelae of degenerative joint disease [15, 16]. Diagnosis is typically achieved through clinical examination and the identification of classic calcific bodies on imaging [17, 18].

Although any synovial joint can be involved, intraarticular chondroid metaplasia occurs most frequently in the knee and hip; the tenosynovium of the metacarpo- and metatarsophalangeal joints, particularly of the flexor tendons, are the most common sites of extraarticular chondromatosis [10, 18–21]. One case of extraarticular synovial chondromatosis involving the pes anserine in association with an osteochondroma has been described, and limited imaging studies (available as online clinical resources) have documented involvement of the SM-MCL bursa; however, detailed reports of tenosynovial chondromatosis of the SM-MCL bursa are lacking [22–24]. Although recurrence is rare after primary excision, it has been associated with malignant transformation in cases of primary synovial chondromatosis [25, 26]. Here we describe the insidious progression and curiously absent pathognomonic imaging results of extraarticular synovial chondromatosis of the SM-MCL bursa.

## Case report

**Fig. 1:**
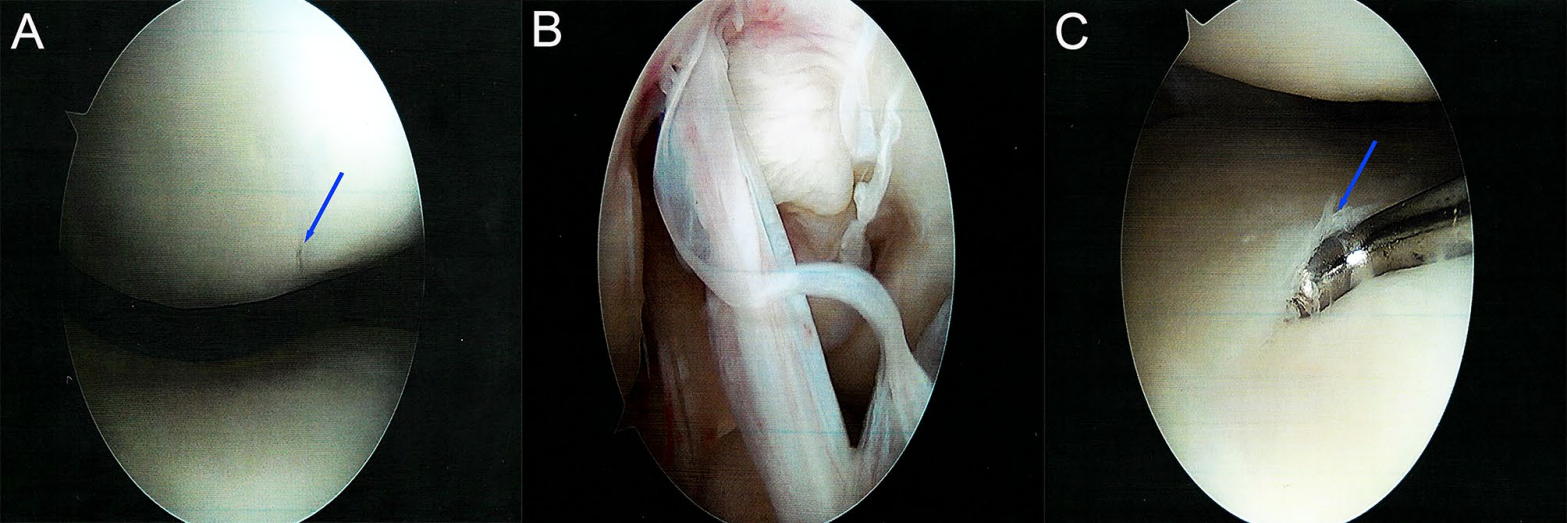
Diagnostic arthroscopic images of the right knee 7 months before presentation. A chondral flap (arrow) was present at the far medial aspect of the medial facet of the patella (A). The intercondylar notch demonstrated hypertrophic synovitis with some tearing of the ligamentum mucosum (B). There was very mild fraying (arrow) at the far posterior root of the lateral meniscus (C)

The patient, a 37-year-old woman, provided written consent to publish the information contained in this case report. She presented with symptoms of progressive right knee pain and stiffness 7 months after undergoing arthroscopic synovectomy of the ligamentum mucosum and medial plica, limited lateral meniscectomy of the posterior root area, and stabilizing chondroplasty of the medial facet of the patella of the ipsilateral knee (Fig. 1). Postoperatively, she had persistent pain in the posteromedial aspect of the knee, an abnormal gait with right knee flexion throughout the stance, and an inability to achieve total knee extension.

**Fig. 2:**
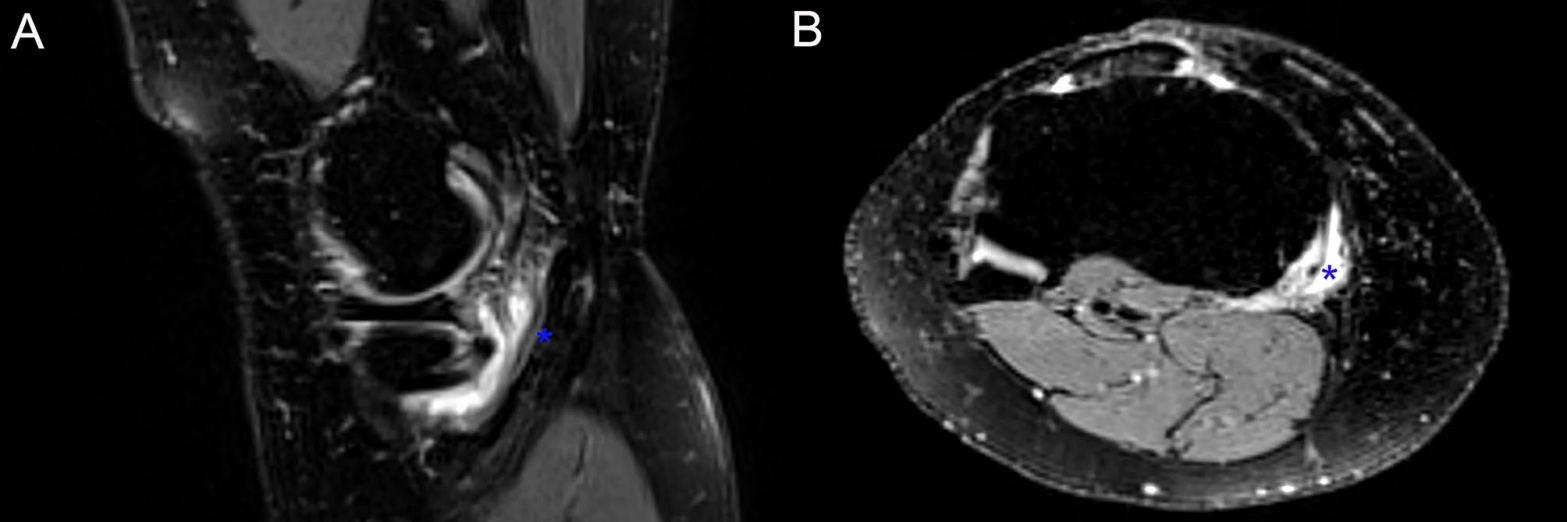
Unenhanced 3-T MRI of the right knee. Focal fluid signal (asterisk) surrounded the distal semimembranosus tendon. The ill-defined margin of the bursa was atypical for simple semimembranosus-medial collateral ligament bursitis (A), and there was mild tendinosis of the semimembranosus tendon, which was otherwise intact (B)

On physical examination, initial inspection of the right knee revealed moderate swelling and fullness of the posteromedial aspect of the joint. There was substantial tenderness over the distal semimembranosus tendon, approximately 2 cm proximal to the insertion site. Right knee active range of motion (AROM) was measured at 0˚ hyperextension, 5˚ extension lag, and 110˚ flexion (0˚/5˚/110˚) (contralateral: 4˚/0˚/135˚). Manual muscle testing of right knee flexion was 4/5 (contralateral: 4+/5) with reported pain. Neurosensory examination was normal, and distal pulses were palpable and bilaterally symmetric. Magnetic resonance imaging (MRI) demonstrated loculated fluid surrounding the distal semimembranosus tendon and a partial, low-grade tear of the distal semimembranosus tendon (Fig. 2).

**Fig. 3:**
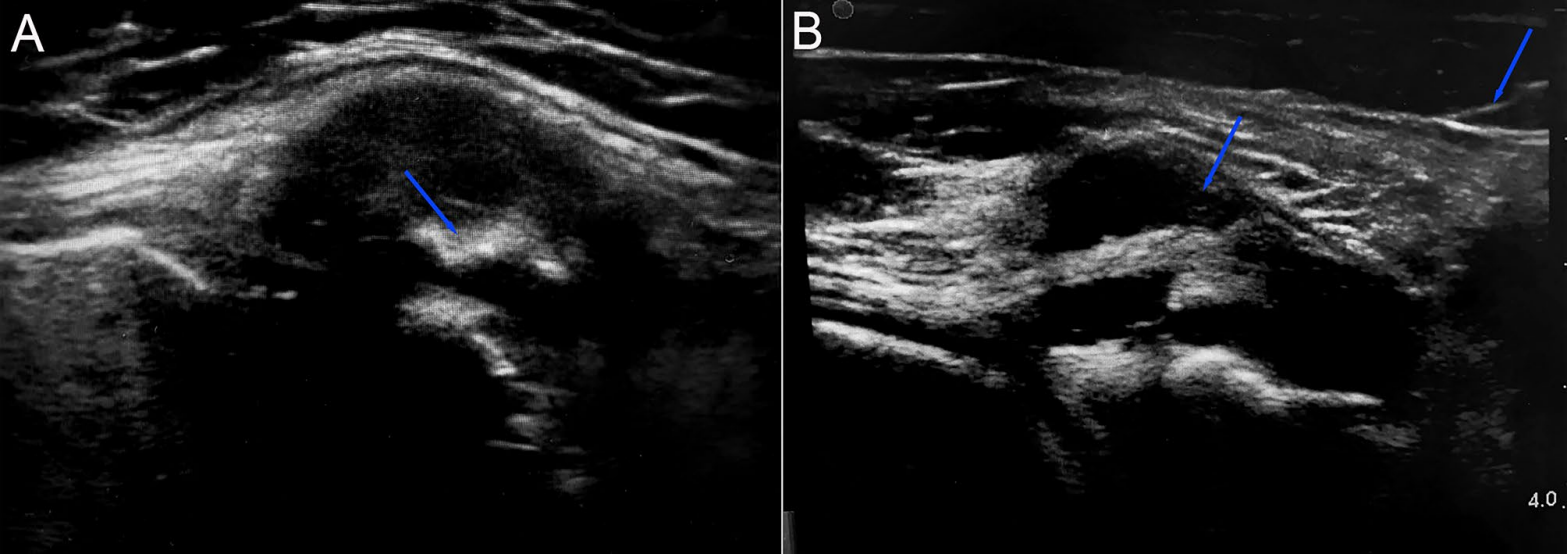
Semimembranosus tendon and bursa visualized with a 12-MHz ultrasonography transducer. Short axis view of the posteromedial knee showing substantial semimembranosus bursitis and hypoechoic swelling surrounding the tendon (arrow) (A) and ultrasound-guided corticosteroid injection of the semimembranosus bursa in long axis view with both proximal and distal extents of the needle indicated (arrows) (B)

Point-of-care ultrasonography demonstrated distal semimembranosus tendon thickening and substantial hypoechoic swelling surrounding the tendon, which correlated with location of maximum pain reported by the patient. An ultrasound-guided injection of triamcinolone (40 mg of Kenalog-40; Bristol-Myers Squibb, New York, NY) into the SM-MCL bursa was performed, providing approximately 2 weeks of improvement in pain (Fig. 3).

One month later, AROM of the right knee was 0˚/2˚/135˚ (contralateral: 3˚/0˚/135˚), and ultrasonography showed persistent semimembranosus tendon thickening and hypoechoic swelling around the tendon and an equivocal semimembranosus tendon tear. The patient agreed to proceed with autologous platelet-rich plasma injection. After injection of 3.5 mL of leukocyte-poor platelet-rich plasma into the semimembranosus tendon sheath and SM-MCL bursa, and 3.0 mL into the joint, there was substantial pain for the first 7–10 days, which was followed by another week of pain relief and mild improvement in knee extension. However, pain and stiffness recurred despite activity modification, gradual strengthening and stretching exercises, and careful attention not to overload the tendon.

**Fig. 4:**
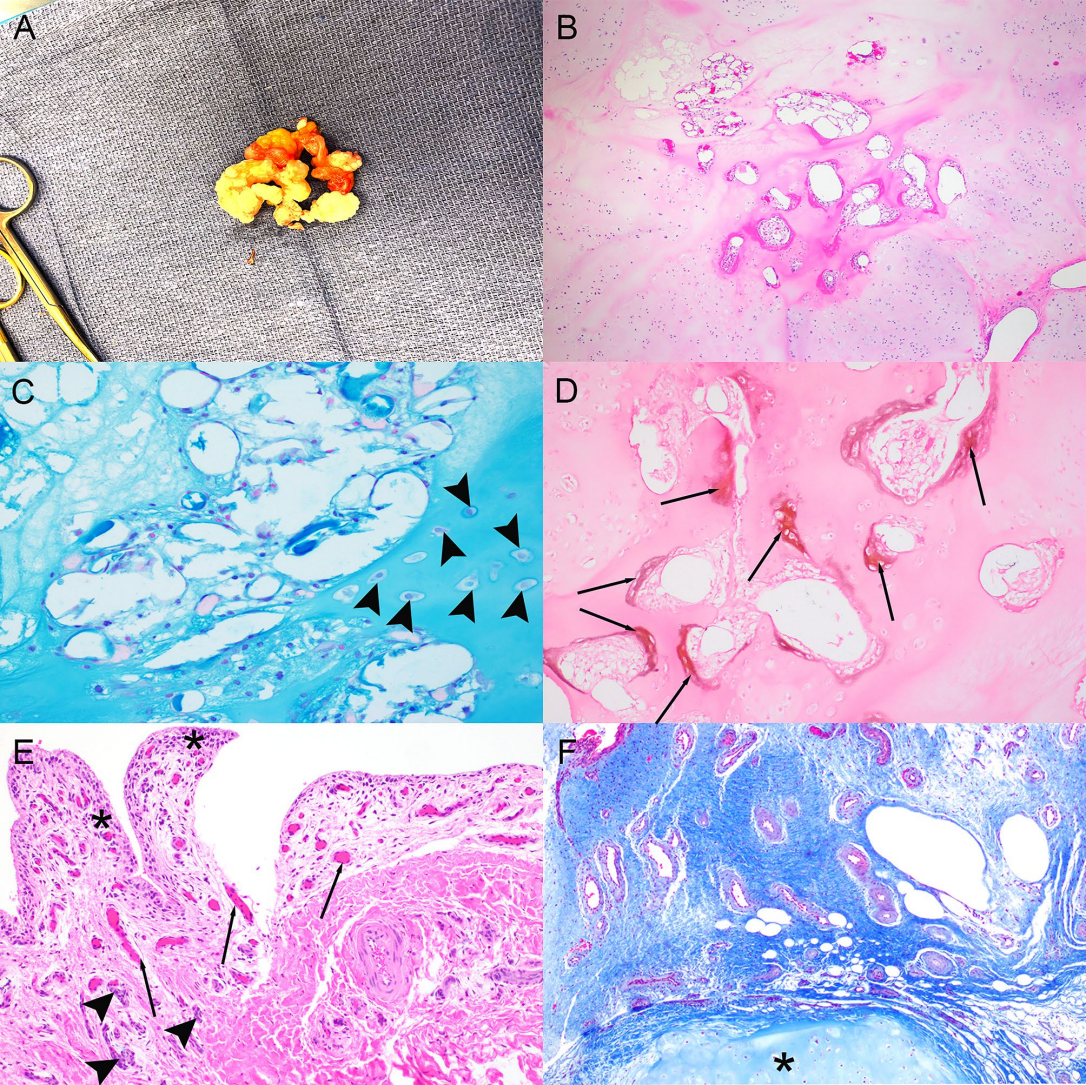
Pathologic appearance of the excised semimembranosus-medial collateral ligament bursal body. The intraoperative *ex situ* gross appearance of the bursa demonstrated multiple fragments of firm, white, nodular and fatty soft tissue. On cut sections, the fragments were either pale, white, and firm or lobulated and yellow (A). Histologically, the tissue was composed primarily of coalescing nodules of mature hyaline cartilage surrounding foci of mineralization (B: hematoxylin and eosin stain, 40× magnification). Higher magnification showing mature chondrocytes chondrocytes within lacunae (arrowheads) surrounded by cartilaginous matrix (blue-green) (C: Alcian blue stain, 200× magnification). The chondroid matrix multifocally surrounded regions of dystrophic mineralization (brown staining; arrows) (D: Von Kossa stain, 100× magnification). The remaining surface synovium consisted of mildly hypertrophied synoviocytes and lymphoplasmacytic inflammation (asterisks) in concert with reactive neovascularization (arrows) (E: hematoxylin and eosin stain, 200× magnification). Mature fibrillar collagen (blue), often in association with small blood vessels, was observed at the periphery of the cartilaginous nodules (asterisk) (F: Masson’s trichrome stain, 40× magnification)

Three months later, because of persistent posteromedial knee pain and swelling and worsening AROM of the affected knee (0˚/14˚/120˚; contralateral: 5˚/0˚/135˚) despite extensive nonoperative treatment, the patient agreed to surgical exploration and SM-MCL bursectomy. A tourniquet was placed around the right upper thigh. The right leg was prepared and draped in a sterile fashion, elevated, and exsanguinated, and the tourniquet pressure was increased to 250 mmHg. An approximately 7-cm-long incision was made at the anteromedial aspect of the knee over the distal medial hamstrings and extending down to the sartorial sleeve. The sleeve was opened, and the medial hamstrings were identified. The SM-MCL bursa was identified and excised. An approximately 2 × 3–cm calcified coral-like body was observed within the bursal excision, and several small (less than 1-cm diameter) bodies were appreciated with further exploration of the bursa, all of which were excised and submitted for pathologic evaluation (Fig. 4).

The wound was irrigated, and with further inspection, confirmation was made that all calcific bodies had been excised. Minimal changes of the tendon were appreciated, and the semimembranosus appeared intact. The sartorial sleeve was left open. The subcutaneous tissue was approximated with 2 − 0 Vicryl suture, and the skin was closed with running subcuticular 3 − 0 Prolene suture and reinforced with wound closure strips. The knee was dressed and immobilized.

Histopathological analysis of the bursal body showed synovial chondromatosis with chronic reactive changes (Fig. 4). Approximately 80% of the tissue had undergone chondroid metaplasia, exhibiting predominantly mature chondrocytes within lacunae arranged in lobules and nests. At the periphery of the cartilaginous nodules were regions of fibrosis and granulation tissue. In addition, the synovium showed reactive changes of hyperplasia and lymphoplasmacytic inflammation.

At initial follow-up, 2 weeks later, no complications were noted, and the patient reported being comfortable. The incision was apposed with minimal swelling and resolving ecchymoses, and the sutures were removed. Right knee AROM was measured to be 0˚/0˚/100˚ with total knee extension achieved for the first time in more than 18 months.

**Fig. 5:**
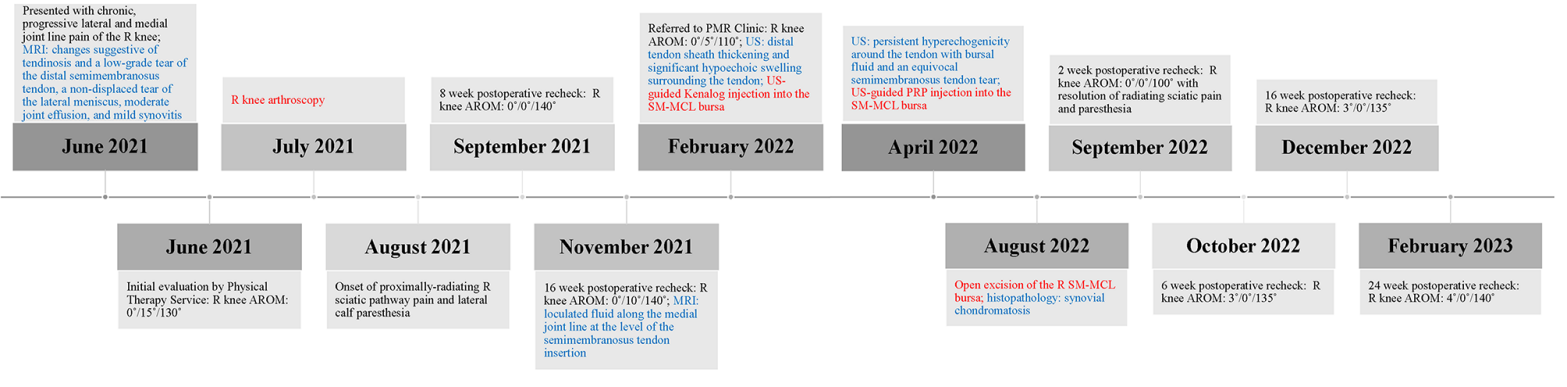
Timeline summarizing diagnostic (blue) and therapeutic (red) details relevant to the clinical course of chronic, progressive semimembranosus-medial collateral ligament bursitis and tenosynovial chondromatosis in our patient. AROM: active range of motion; R: right; MRI: magnetic resonance imaging; PMR: physical medicine and rehabilitation; PRP: platelet-rich plasma; US: ultrasound

The patient resumed physical therapy to mobilize the soft tissues and joint, focusing on gradual restoration of range of motion. At 6 weeks postoperatively, AROM was 3˚/0˚/135˚ (contralateral: 5˚/0˚/135˚), and a favorable outcome was achieved for the patient. Good rehabilitation potential and response to therapy were documented in the physical therapists’ subjective assessments throughout the course of treatment, and tolerability of intervention with regard to fatigue, pain, and soreness was assessed using the visual analog scale [27]. The patient also demonstrated ongoing receptiveness to the treatment plan by following therapists’ advice and undertaking prescribed exercises during more than 65 h of in-clinic attendance over 16 months. A timeline detailing relevant diagnostic and therapeutic interventions from the time of presentation through the most recent follow-up evaluation is provided below (Fig. 5).

## Discussion

Diagnosis of synovial chondromatosis relies primarily on patient history and clinical examination findings, including joint pain, swelling, and restricted range of motion, as well as typical associated imaging findings, including the presence of calcified intra-articular bodies detectable on conventional radiographs. Sonographic characteristic of synovial chondromatosis may appear as multiple small hyperechoic foci with posterior acoustic shadowing, and thickened synovium [28]. Power Doppler will reveal absent hyperemia to suggest avascularity [29]. In the absence of calcification, MRI can demonstrate unmineralized bodies and synovial nodules with signal characteristics of cartilaginous tissue (i.e., T-1 intermediate to low signal and T-2 high signal). In the presence of calcification, foci of low signal within these nodules are detectable in all sequences and can be accentuated in gradient echo sequences because of blooming artifacts [30–32]. In addition, using advanced imaging modalities such as computed tomography and MRI to precisely localize extraarticular lesions has proven beneficial in the noninvasive diagnosis of tenosynovial chondromatosis [13, 18].

Grossly, these lesions consist of multilobulated nodules of hyperplastic synovium with metaplasia to hyaline cartilage [13]. Typically, histopathological analysis correlates well with imaging studies and routinely confirms chondroid metaplasia, regressive calcification, and loose fibrous connective tissue admixed with hyperplastic synoviocytes in excised surgical specimens [13, 17, 18, 33–35]. Infiltration by chronic inflammatory cells and the presence of hyperemic vessels are also commonly observed on histopathologic evaluation of synovial chondromatosis [13, 16, 36].

## Conclusion

Given this patient’s history of initially lateral, then medial, joint line pain early in the course of symptoms, degenerative arthropathy and resulting mechanical strain caused by tears in the lateral meniscus and ligamentum mucosum are considered to be the nidus for promoting a proinflammatory milieu for the semimembranosus tendon and SM-MCL bursa and the subsequent development of synovial chondromatosis. A partial, low-grade tear at the distal semimembranosus tendon attachment was also a likely contributor to this process, and semimembranosus tendonitis following total knee arthroplasty has been documented, so it is reasonable to conclude that tendinopathy and SM-MCL bursitis may have occurred as sequelae to prior ipsilateral arthroscopy and chondroplasty, with the chronic inflammation ultimately inciting chondroid metaplasia [37]. However, our inability to definitively identify the precedent cause of chondromatosis in this patient represents the primary limitation of this case report. Interestingly, although this case was confirmed through histopathologic evaluation of the excised bursal body, radiographs, MRI, and ultrasonography failed to identify the calcific bodies typical of the disease. Although the discordance between imaging and pathology in this case remains unresolved, lesion localization specifically to the small SM-MCL bursa and obfuscation of the bursa by loculated peritendinous fluid, typical of SM-MCL bursitis, are considered most contributory in this case. Further, the intralesional multifocal mineralization observed on histopathological analysis was relatively small, with foci measuring no more than 250 μm in their greatest dimension, likely exceeding the resolution sensitivity of the 3-T magnet used for the imaging study in this report. The patient experienced a favorable outcome after SM-MCL bursectomy for the treatment of synovial chondromatosis.

Commitment to a skilled physical therapy program and judicious monitoring for recurrence should facilitate uncomplicated return to normal function and pain-free activity in these cases. This report highlights that synovial chondromatosis should be included in the differential diagnosis for insidious knee pain and recalcitrant SM-MCL bursitis, even in the absence of supporting imaging.

## Data Availability

Data sharing is not applicable to this article as no datasets were generated or analyzed during the current study.

## Abbreviations

AROM: Active range of motion
MRI: Magnetic resonance imaging
